# Intestinal Angioedema Induced by Angiotensin-Converting Enzyme Inhibitors: A Diagnosis of Exclusion

**DOI:** 10.7759/cureus.99642

**Published:** 2025-12-19

**Authors:** Rita Pera, Sara Sá, João Lagarteira, Andrei Gradinaru, Ana Figueiredo

**Affiliations:** 1 Internal Medicine Department, Unidade Local de Saúde do Nordeste, Bragança, PRT

**Keywords:** abdominal pain, adverse drug reactions, angioedema, angiotensin-converting enzyme inhibitors, enterocolitis, small bowel angioedema

## Abstract

Angiotensin-converting enzyme inhibitor (ACEI)-induced intestinal angioedema is a rare and frequently underdiagnosed condition, often presenting with nonspecific gastrointestinal symptoms that can mimic inflammatory, infectious, or neoplastic diseases. We report the case of a 42-year-old female patient who recently started perindopril (an ACEI) for uncontrolled hypertension and developed recurrent abdominal pain and diarrhea. Extensive laboratory and imaging workup, complemented by endoscopic and histological evaluations, did not identify any alternative etiology. Symptoms resolved completely after discontinuation of perindopril, confirming ACEI-induced intestinal angioedema. Early recognition of this entity is essential to avoid invasive procedures and ensure complete resolution with simple drug discontinuation.

## Introduction

Angiotensin-converting enzyme inhibitors (ACEIs) are widely prescribed for hypertension, heart failure, and other cardiovascular conditions. Angioedema is a recognized adverse effect, occurring in approximately 0.1-0.7% of treated patients, most commonly involving the lips, tongue, periorbital region, or upper airway [1,2]. Intestinal involvement is infrequently recognized and presents with abdominal pain, nausea, vomiting, and diarrhea, and can mimic inflammatory, infectious, or neoplastic diseases. Delayed recognition can lead to repeated emergency visits and extensive diagnostic workups, sometimes prompting unnecessary invasive procedures [1,3-6].

The underlying pathophysiology is primarily mediated by accumulation of bradykinin, a potent vasoactive peptide normally degraded by ACE. Inhibition of ACE results in elevated bradykinin levels, which bind to B2 receptors, activate downstream nitric oxide and prostaglandin pathways, and increase endothelial permeability, producing submucosal edema in the bowel wall [7-9]. Additional mediators, including des-Arg9-bradykinin (acting via B1 receptors) and substance P, may further amplify vascular leakage under inflammatory conditions [7,8]. Unlike histamine-mediated angioedema, ACEI-induced reactions typically lack urticaria or eosinophilia [1,2,10].

Beyond ACE inhibition, impairment of alternative bradykinin-degrading pathways also appears to modulate individual susceptibility. Reduced activity of aminopeptidase P (APP) and dipeptidyl peptidase-IV (DPP-IV) has been associated with a heightened risk of ACEI-induced angioedema [9,11], and genetic variants of the XPNPEP2 gene (encoding APP) have been linked to increased vulnerability in genome-wide studies [12]. These factors may explain why only a small proportion of ACEI users develop angioedema despite widespread exposure.

Clinically, intestinal angioedema presents with nonspecific symptoms such as abdominal pain, nausea, vomiting, and diarrhea, often accompanied by ascites. Contrast-enhanced computed tomography (CT) is particularly informative, typically demonstrating segmental circumferential bowel wall thickening with submucosal edema (“target sign”), mucosal hyperenhancement, and free peritoneal fluid, with complete resolution following ACEI discontinuation [3-6,10]. Because no specific biomarker exists, ACEI-induced intestinal angioedema remains a diagnosis of exclusion, requiring recognition of the temporal association with ACEI therapy and clinical or radiologic resolution after drug withdrawal [1-6]. Early identification is crucial to prevent unnecessary investigations, interventions, and prolonged hospitalizations.

We report a case of ACEI-induced intestinal angioedema in a 42-year-old female patient with recurrent abdominal symptoms, in whom clinical and radiologic normalization occurred promptly after withdrawal of perindopril. This case underscores the importance of early recognition to prevent unnecessary hospitalizations, diagnostic procedures, and therapeutic interventions.

## Case presentation

A 42-year-old female patient with a history of hypertension, dyslipidemia, overweight, and hypothyroidism was started on perindopril 8 mg/day due to persistent blood pressure elevation despite lifestyle optimization. At that time, her chronic medication consisted only of levothyroxine 125 µg/day and atorvastatin 20 mg/day, with no recent initiation of other drugs. Approximately three weeks after starting perindopril, she developed diffuse abdominal pain and profuse diarrhea (>10 bowel movements/day), without fever, gastrointestinal bleeding, vomiting, or weight loss. She presented multiple times to the emergency department with only transient relief after symptomatic therapy.

On admission to the emergency department, she was hemodynamically stable (blood pressure 123/62 mmHg, heart rate 87 bpm, SpO₂ 98% on room air) and afebrile. She appeared well perfused and hydrated, without jaundice or cyanosis. Abdominal examination revealed a soft but diffusely tender abdomen on deep palpation, with no signs of peritoneal irritation. No peripheral or oropharyngeal angioedema was noted.

**Figure 1:**
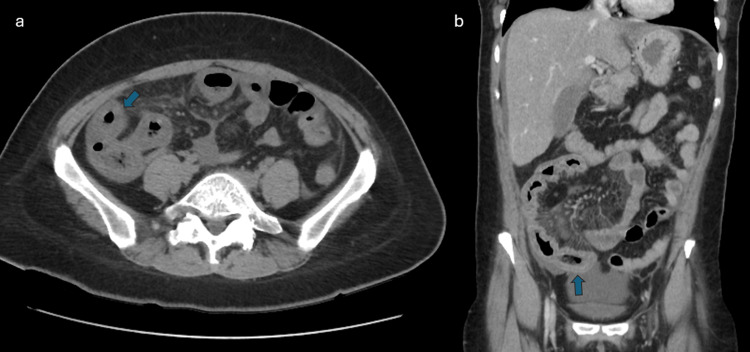
First contrast-enhanced computed tomography (CT) scan of the abdomen and pelvis In the axial (a) and coronal sections (b), we can see significant diffuse thickening of the distal ileum (arrows).

An initial computed tomography (CT) scan of the abdomen and pelvis, performed five weeks after symptom onset, revealed significant diffuse thickening of the distal ileum and ileocecal valve region, extending over a long area, associated with mesenteric vascular engorgement, surrounding densification, and moderate volume of peritoneal effusion in all quadrants (Figure 1), prompting referral to Gastroenterology.

Laboratory findings are summarized in Table 1. Complete blood count and coagulation tests were within normal limits, as were electrolytes, renal function, and liver enzymes. Inflammatory markers showed no significant elevation. Thyroid function and immunological studies, including serum protein electrophoresis, were unremarkable. Serologic testing for celiac disease (tissue transglutaminase IgA and endomysial antibodies) and anti-Saccharomyces cerevisiae antibodies (ASCAs) was negative.

**Table 1:** Laboratory test results on admission “–" indicates no normal reference range is applicable ALP, alkaline phosphatase; ALT, alanine transaminase; ANA, antinuclear antibodies; ANCA, autineutrophil cytoplasmic antibody; ASCA, anti-saccharomyces cerevisiae antibodies; AST, aspartate aminotransferase; CK, creatine kinase; CRP, C-reactive protein; ESR, erythrocyte sedimentation rate; GGT, gamma-glutamyl transferase; HBV, hepatitis B virus; HCV, hepatitis C virus; HIV, human immunodeficiency virus; INR, international normalized ratio; LDH, lactate dehydrogenase; MPO, myeloperoxidase; PR3, proteinase 3; TSH, thyroid-stimulating hormone; tTG, tissue transglutaminase; T4, thyroxine

Parameter	Result	Normal Range
Hemoglobin (g/dL)	14.4	12.3-15.3
Total leucocyte count (x10^9^/L)	11.21	4.4-11.3
Differential leucocyte count (%)		
Neutrophils	70.2	50-70
Lymphocytes	25.8	25-40
Monocytes	5.6	2-8
Eosinophils	1.1	1-4
ESR (mm)	20	4.0-11.0
Platelet count (x10^9^/L)	277	150-450
Sodium (mEq/L)	137	137-145
Potassium (mEq/L)	3.6	3.5-5.1
Chloride (mEq/L)	101	98-107
Urea (mg/dL)	32	17-43
Creatinine (mg/dL)	0.8	0.66-1.09
ALT (U/L)	33	<34
AST (U/L)	28	<31
Total bilirubin (mg/dL)	0.57	0.3-1.2
Direct bilirubin (mg/dL)	0.14	<0.2
ALP (U/L)	58	30-120
GGT (U/L)	26	<38
LDH (U/L)	152	<248
CK (U/L)	54	<145
INR	0.97	-
CRP (mg/dL)	0.25	<0.1
TSH (uUI/mL)	1.06	0.35-4.94
Free T4 (ng/dL)	1.23	0.7-1.48
Immunoglobulin A (g/L)	1.23	0.7-4.0
Immunoglobulin G (g/L)	8.52	7.0-16
Immunoglobulin M (g/L)	1.56	0.4-2.3
Complement C3 (g/L)	1.51	0.9-1.8
Complement C4 (g/L)	0.32	0.1-0.4
HIV	Negative	-
HBV	Negative	-
HCV	Negative	-
ANA	1/80	<1/160
ANCA MPO (UI/mL)	2.1	<3.5=Negative
ANCA PR3 (UI/mL)	1.2	<2.0=Negative
tTG antibodies IgA (U/mL)	0.2	<7=Negative
Endomysial antibodies IgA	Negative	<1/5=Negative
ASCA IgA (U/mL)	Negative	<20=Negative
ASCA IgG (U/mL)	Negative	<20=Negative

Stool studies were negative for bacterial, viral, and parasitic pathogens, including *Clostridium difficile* antigen and toxins. Fecal calprotectin and pancreatic elastase were within normal ranges. Upper and lower endoscopy revealed no significant abnormalities, and colon biopsies demonstrated preserved mucosal architecture with minimal nonspecific chronic inflammation.

**Figure 2:**
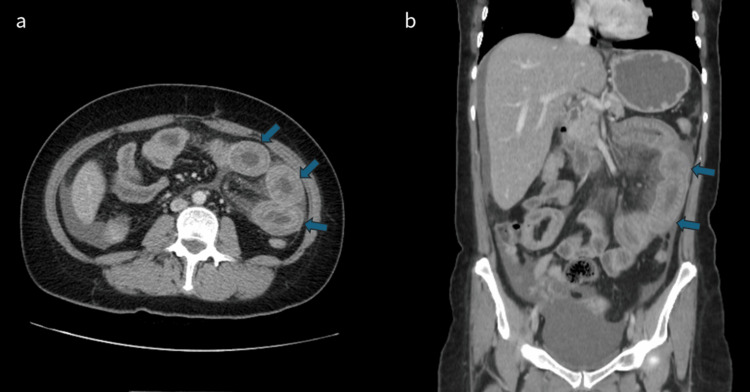
Second contrast-enhanced computed tomography (CT) scan of the abdomen and pelvis. In the axial (a) and coronal section (b), we can see circumferential wall thickening of the proximal jejunum (arrows) with submucosal edema and mucosal hyperenhancement.

Two months after symptom onset, she returned to the emergency department with worsening abdominal pain and ongoing diarrhea. A repeat abdominal CT scan showed circumferential wall thickening of the proximal jejunum with submucosal edema and mucosal hyperenhancement, suggestive of enteritis (Figure 2).

**Figure 3:**
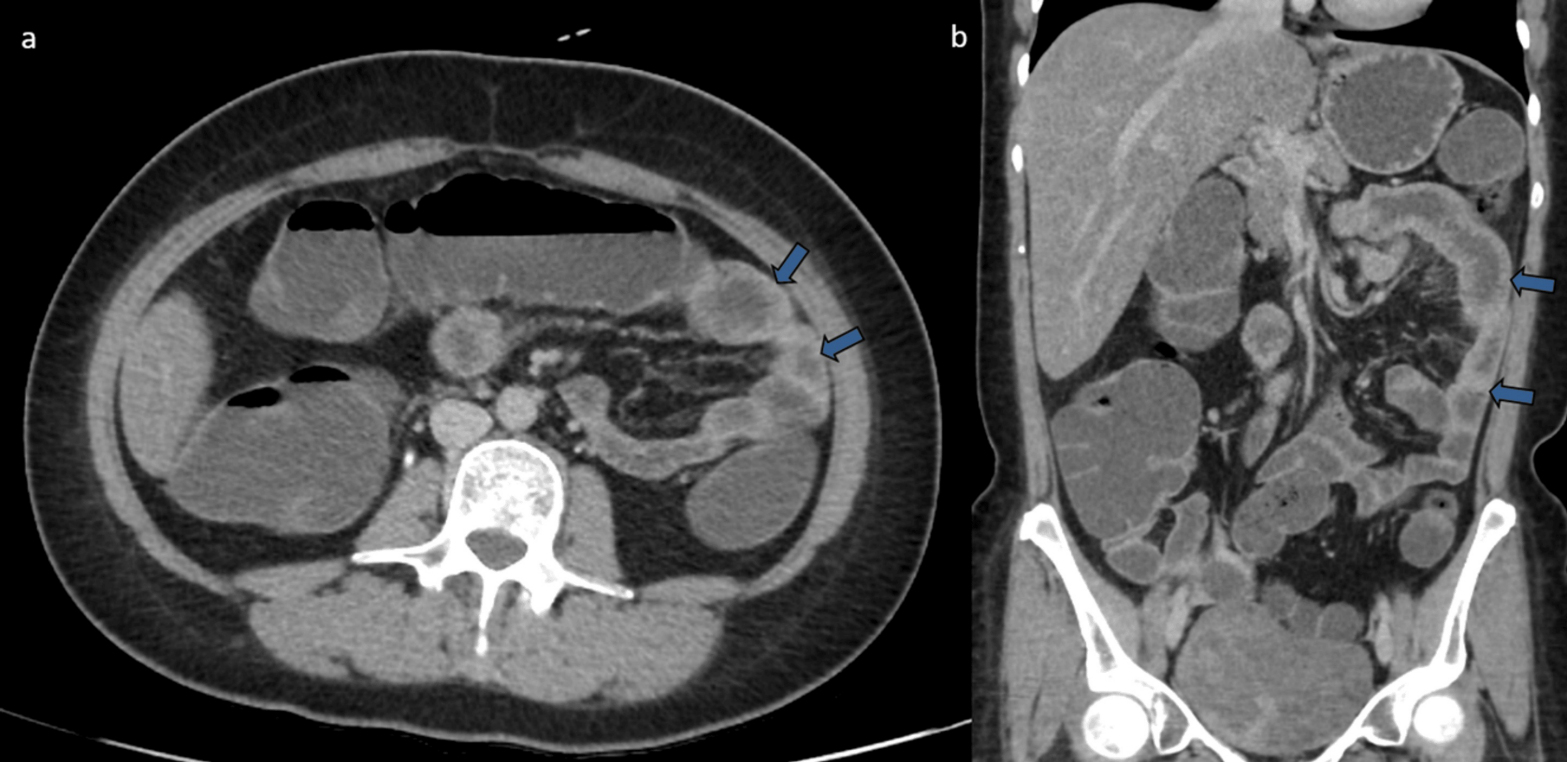
Computed tomography (CT) enterography performed seven days after angiotensin-converting enzyme inhibitor suspension. In the axial (a) and coronal section (b), we can see normal distensibility of jejunal and ileal loops, without dilated or thickened small bowel segments (arrows). No peritoneal effusion.

**Figure 4:**
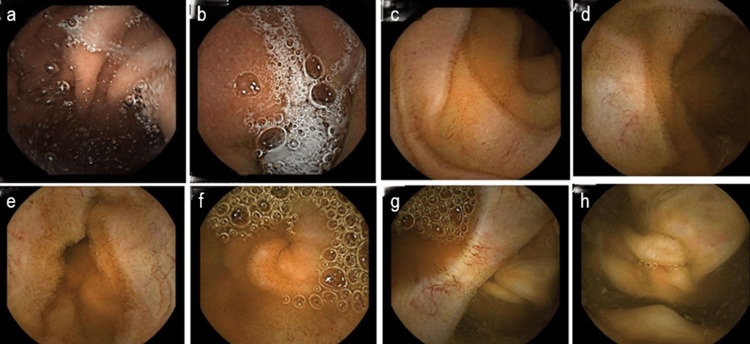
Capsule endoscopy showed no abnormalities. Capsule endoscopy showed no abnormalities. Images are displayed in chronological sequence of capsule transit: image a shows the gastric lumen, whereas image h depicts the cecum, marking the end of small-bowel evaluation

She was admitted for further investigation, and perindopril was discontinued. Diagnostic paracentesis revealed serous ascitic fluid containing mesothelial cells and rare atypical lymphocytes. Flow cytometry and immunophenotyping of the fluid showed no evidence of malignancy. An enterography CT performed seven days later was completely normal (Figure 3), and capsule endoscopy also showed no abnormalities (Figure 4).

Given the exclusion of infectious, inflammatory, and neoplastic causes, and the complete clinical and radiological resolution following withdrawal of the ACE inhibitor, a diagnosis of ACEI-induced intestinal angioedema was established. The patient remained asymptomatic at discharge and has had no recurrence to date. Her antihypertensive therapy was switched to a calcium-channel blocker, with adequate blood pressure control during follow-up.

## Discussion

ACE inhibitor-induced intestinal angioedema remains underrecognized because its clinical and imaging features overlap significantly with more common inflammatory, infectious, and neoplastic disorders [1-6]. In our case, the temporal association between perindopril initiation and the onset of symptoms, combined with characteristic imaging findings and rapid resolution after drug withdrawal, proved central to establishing the diagnosis.

Our patient presented with diffuse abdominal pain and diarrhea shortly after starting perindopril. CT imaging showed segmental small bowel wall thickening with submucosal edema and mild ascites, findings compatible with intestinal angioedema but also seen in Crohn’s disease, vasculitis, infection, or neoplastic processes [1-4]. The apparent variation in affected bowel segments between imaging studies likely reflects the transient and reversible nature of bradykinin-mediated angioedema rather than progressive segmental disease [1,2,4].

The differential diagnosis was carefully explored. Infectious and inflammatory markers were not supportive of active inflammatory bowel disease or systemic vasculitis, and endoscopic biopsies did not reveal features of Crohn’s disease. Cytological evaluation of the ascitic fluid showed no diagnostic abnormalities, and flow cytometry with immunophenotyping excluded a clonal lymphoid population, thereby ruling out malignancy. The complete normalization of imaging one week after ACE inhibitor discontinuation was a decisive finding, strengthening the causal relationship.

Because no specific biomarker exists, the diagnosis is ultimately one of exclusion, guided by clinical suspicion and confirmed by reversibility after stopping the ACE inhibitor. Early recognition is essential to prevent unnecessary invasive procedures and avoid delays in management [1-6].

Re-exposure to ACE inhibitors is contraindicated due to the known risk of recurrence [1,2]. Although cross-reactivity with angiotensin receptor blockers (ARBs) is uncommon, it has been reported, and these agents should be used cautiously [1-3]. In this case, the patient was transitioned to a calcium-channel blocker, achieving adequate blood pressure control without recurrence.

## Conclusions

ACEI-induced intestinal angioedema is an underrecognized cause of recurrent abdominal pain and diarrhea that can mimic inflammatory or infectious conditions, often leading to unnecessary investigations. In this case, timely recognition and withdrawal of perindopril led to complete resolution of symptoms and imaging abnormalities, confirming the diagnosis. This report highlights the diagnostic challenges of this entity and underscores the importance of maintaining a high index of suspicion in patients recently started on ACEIs, illustrating how careful evaluation can prevent invasive procedures and ensure effective management.
